# Commentary: The Need for Bayesian Hypothesis Testing in Psychological Science

**DOI:** 10.3389/fpsyg.2017.01434

**Published:** 2017-08-23

**Authors:** Jose D. Perezgonzalez

**Affiliations:** Business School, Massey University Palmerston North, New Zealand

**Keywords:** *p*-value, logic, reductio argument, modus tollens, data testing, statistics

A commentary on **The Need for Bayesian Hypothesis Testing in Psychological Science** *by Wagenmakers, E. J., Verhagen, J., Ly, A., Matzke, D., Steingroever, H., Rouder, J. N., et al. (2017). Psychological Science Under Scrutiny: Recent Challenges and Proposed Solutions, eds S. O. Lilienfeld and I. D. Waldman (Chichester: JohnWiley & Sons), 123–138.*

Wagenmakers et al. (2017) argued the need for a Bayesian approach to inferential statistics in *Psychological Science under Scrutiny*. Their primary goal was to demonstrate the illogical nature of *p*-values, while, secondarily, they would also defend the philosophical consistency of the Bayesian alternative. In my opinion, they achieved their secondary goal but failed their primary one, thereby this contribution. I will, thus, comment on their interpretation of the logic underlying *p*-values without necessarily invalidating their Bayesian arguments.

Historical criticisms (e.g., Harshbarger, 1977, onwards) have already delved in the illogical nature of null hypothesis significance testing (NHST)—a mishmash of Fisher's, Neyman-Pearson's, and Bayes's ideas (e.g., Gigerenzer, 2004; Perezgonzalez, 2015a). Wagenmakers et al.'s original contribution is to generalize similar criticisms to the *p*-value itself, the statistic used by frequentists when testing research data.

Wagenmakers et al. assert that Fisher's disjunction upon obtaining a significant result—i.e., either a rare event occurred or H_0_ is not true (Fisher, 1959)—follows from a logically consistent *modus tollens* (also Sober, 2008): If P, then Q; not Q; therefore not P, which the authors parsed as, If H_0_, then not y; y; therefore not H_0_.

“Y” is defined as “the observed data…[summarized by] the *p*-value” (p. 126). Therefore, their first premise proposes that, if H_0_ is true, the observed *p*-values cannot occur (also Cohen, 1994; Beck-Bornholdt and Dubben, 1996). This seems incongruent, as the first premise of a correct *modus tollens* states a general rule—H_0_ implies “not y”—while the second premise states a specific test to such rule—“this y” has been observed. If the authors meant for “y” to represent “significant data” as a general category in the first premise and as a specific realization in the second, a congruent *modus tollens* would ensue, as follows (also Pollard and Richardson, 1987):

If H_0_, then not *p* < sig; *p* < sig (observed); therefore not H_0_      (1)

Wagenmakers et al.'s (also Pollard and Richardson, 1987; Cohen, 1994; Falk, 1998) main argument is that a correct *modus tollens* is rendered inconsistent when made probabilistic, as follows:

If H_0_, then *p* < sig very unlikely; *p* < sig; therefore probably not H_0_      (2)

There are, however, three problems with (2), problems which I would like to comment upon. One problem is stylistic: The first premise states a redundant probability; that is, that a significant result—which already implies an unlikely or improbable event under H_0_—is unlikely. Therefore, the syllogism could be simplified as follows:

If H_0_, then *p* < sig; *p* < sig; therefore probably not H_0_      (3)

Correction (3) now highlights another of the problems: The second premise simply affirms that an unlikely result just happened (also Cortina and Dunlap, 1997), something which is neither precluded by the first premise (no contrapositive ensues; Adams, 1988) nor formally conducive to a logical conclusion under *modus tollens* (Evans, 1982). Indeed, in the examples given (also by Cohen, 1994; Beck-Bornholdt and Dubben, 1996; Cortina and Dunlap, 1997; Krämer and Gigerenzer, 2005; Rouder et al., 2016), Tracy is a US congresswoman, Francis is the Pope, and John made money at the casino, each despite their odds against. Yet, none of those realizations deny the consequents. A correction, following Harshbarger (1977) and Falk (1998), would state:

If H_0_, then not *p* < sig; *p* < sig; therefore probably not H_0_      (4)

Correction (4) brings to light the most important problem: *Modus tollens* is in the form, If P, then Q; not Q; therefore not P. Thus, whenever the consequent (Q) gets denied in the second premise, it leads to denying the antecedent (P) in the conclusion. Such operation ought to prevail with probabilistic premises, as well (e.g., Oaksford and Chater, 2001, 2009; Evans et al., 2015), whereby a probable consequent (Q_*p*_) may be denied without its probability warranting transposition onto a non-probabilistic antecedent (P). For example, if all red cars (P) have a 95% chance of getting stolen (*Q* ≥ 0.95) and we learn of a Lamborghini with a lesser probability of so disappearing (not *Q* ≥ 0.95), it is logical to conclude that the Lamborghini is not red (not P).

In comparison, Bayesian logic allows for the antecedent to be probable. For example, if John always submits to Nature (Q) whenever his subjective probability of getting published soars above 20% (*P* > 0.2), yet he is not submitting his latest article (not Q), it is logical to conclude that he probably expects no publication (not *P* > 0.2).

We can, thus, envisage P or Q, or both, as probable without either warranting inter-transposition of their probabilities, which brings us back to a valid *modus tollens* (1). Said otherwise, while Bayesian statistics allow for the antecedent to be probable (P_p_), Fisher's and Neyman-Pearson's tests assume exact antecedents (P); therefore, a probabilistic conclusion does not hold with frequentist tests (Mayo, 2017).

It ought to be noted that the *p*-value is a statistic descriptive of the probability of the data under H_0_ [p(D|H_0_)] (Perezgonzalez, 2015b). The *reductio ad absurdum* argument may be informed by, but it is not dependent on, such *p*-value, the *reductio* being determined exclusively by the chosen level of significance. For “it is open to the experimenter to be more or less exacting in respect of the smallness of the probability he would require before he would be willing to admit that his observations have demonstrated a positive result. It is obvious that an experiment would be useless of which no possible result would satisfy him” (Fisher, 1960, p.13).

In conclusion, the technology of frequentist testing holds their *modus tollens* logically. Wagenmakers et al.'s criticism of the *p*-value is faulty in that they allow for a probability transposition not warranted either by *modus tollens* or by the technical apparatus of Fisher's and of Neyman-Pearson's tests. This critique, however, does not extend to their Bayesian argumentation, an approach much needed for testing hypotheses—rather than just testing data—in contemporary science.

## Author contributions

The author confirms being the sole contributor of this work and approved it for publication.

### Conflict of interest statement

The author declares that the research was conducted in the absence of any commercial or financial relationships that could be construed as a potential conflict of interest.
